# Chitosan Spray-Dried Microparticles for Controlled Delivery of Venlafaxine Hydrochloride

**DOI:** 10.3390/molecules22111980

**Published:** 2017-11-15

**Authors:** Inmaculada Aranaz, Ines Paños, Carlos Peniche, Ángeles Heras, Niuris Acosta

**Affiliations:** 1 Department of Physical Chemistry II. Pharmacy Faculty, Biofuncional Studies Institute, Complutense University, Paseo Juan XXIII, 1, 28040 Madrid, Spain; iaranaz@hotmail.com (I.A.); inespanos@hotmail.com (I.P.); aheras@ucm.es (Á.H.); 2 Center of Biomaterials, University of Havana Ave. Universidad s/n entre G y Ronda, Vedado, 10400 La Habana, Cuba; cpeniche2015@yahoo.com

**Keywords:** microcapsules, modelling, functional characterization, venlafaxine, drug release

## Abstract

Venlafaxine controlled drug delivery systems using different matrixes have been tested to reduce undesirable side effects in the treatment of depression. The legal status of chitosan (Cs) in Pharmacy has dramatically improved after its acceptance as excipient in several *Pharmacopeias* and, therefore, there is great interest in pharmaceutical formulations based on this polymer. In this paper, chitosan microcapsules cross-linked with sodium tripolyphosphate (TPP) for oral delivery of venlafaxine were formulated using the spray drying technique. The effect of chitosan physico-chemical properties, TPP concentration and TPP/Cs ratio on drug release was evaluated. The microcapsules were characterized in terms of size, zeta potential and morphology. The physical state of the drug was determined by X-ray diffraction (XRD) and the drug release from the microcapsules was studied in simulated gastric and intestinal fluids. The release pattern fitted well to the Peppas-Koersmeyer model with n exponents indicating anomalous transport.

## 1. Introduction

Depression is a mental disorder, characterized by persistent sadness and a loss of interest in activities that the patient normally enjoys, accompanied by an inability to carry out daily activities, for at least two weeks. This is a recurrent and chronic disorder requiring long-term treatment. Depression treatment has a multidisciplinary approach which includes patient education and support, evaluation of suicide risk and treatment of symptoms with antidepressant medication, psychotherapy, or both. Patients with severe depression or melancholic symptoms may respond better to tricyclic antidepressants such as venlafaxine [1]. Venlafaxine hydrochloride is a highly water soluble, non-tricyclic antidepressant agent of the serotonin-norepinephrine reuptake inhibitor (SNRI) class. The steady state half-lives of venlafaxine and *o*-desmethylvenlafaxine (active metabolite of venlafaxine) are 5 and 11 h, respectively, necessitating the administration, two or three times daily so as to maintain adequate plasma levels of the drug [2]. Moreover, immediate-release formulations have many side-effects, including nausea, insomnia, weakness, drowsiness and constipation [3]. Therefore, venlafaxine hydrochloride is a good candidate to be formulated in controlled drug delivery systems. Not only will this formulation reduce the number of doses but it also might reduce side-effects. Several venlafaxine formulations for controlled release of the drug via different adsorption routes can be found in the literature. For instance, montmorillonite–polyvinylpyrrolidone, montmorillonite-PLGA composites and montmorillonite-alginate microspheres have been designed for oral controlled delivery of venlafaxine [3,4,5]. Venlafaxine buccal tablets produced by compression of mucoadhesive polymers such as hydroxypropylmethylcellulose K4M (HPMC K4M), carboxymethylcellulose (CMC), carbopol 934P and chitosan were reported. Among the cited polymers the best results were observed with hydroxypropylmethylcellulose [6]. The same buccal route has been used to deliver venlafaxine from chitosan-polyvinyl pyrrolidone films and chitosan-beta glycerol phosphate thermo-responsible gels [7,8].

Chitosan is a natural, biocompatible, and biodegradable biopolymer derived from chitin [9]. Chemically, chitosan is a copolymer of *N*-acetylglucosamine and glucosamine units. It cannot be defined as a unique compound and its properties mainly depend on its molecular weight and deacetylation degree (DD), usually expressed as a molar percent of the d-glucosamine unit relative to the total D-glucosamine and N-acetyl-D-glucosamine units [10]. Due to the presence of primary amino groups in the polymer chain, chitosan is soluble in acidic aqueous solution at pH below its p*K*_a_ (6.1–6.5) [11]. The regulatory status of chitosan in Pharmacy has improved after chitosan hydrochloride approval as excipient by the European Pharmacopeia and more recently, chitosan has since been approved as excipient by the US Pharmacopeia [12,13]. From a technological point of view, chitosan is a versatile polymer, and chitosan based drug delivery systems using chitosan films, scaffolds, nanoparticles and microparticles have been reported. Spray drying is widely used in the pharmaceutical industry for the preparation of microparticles [14]. Spray drying is a one-stage continuous process, easy to scale-up, and only slightly dependent upon solubility of drug and polymer. Moreover, it can be used with heat resistant or heat-sensitive drugs, water soluble and water-insoluble drugs and for hydrophilic or hydrophobic polymers [15].

Since chitosan is a hydrophilic polymer and swells in aqueous media, mainly when the polymer is in its cationic state, polymer cross-linking is desirable to obtain a drug controlled release from chitosan spray-dried microcapsules [16]. Typical chitosan cross-linking with dialdehydes such as glutaraldehyde may induce undesirable effects and for biomedical applications ionic cross-linkers such as tripolyphosphate (TPP) are preferred [17]. TPP is classified by the Food and Drug Administration (FDA) as being a Generally Recognized as Safe Substance (GRAS) [18]. Moreover, it has been approved as a food additive, both in the USA and in Europe, in a wide variety of foods [19].

Chitosan has shown mucoadhesive properties both in vitro and in vivo in several studies both in animals and humans [20,21,22]. This property is retained in chitosan microspheres. Therefore, chitosan microspheres as vehicle for drug delivery are of interest since mucoadhesivity enlarges the drug residence time at the absorption site creating a concentration gradient which improves drug absorption. Interestingly, chitosan exhibits adsorption promotor activity due to its ability to open the tight junctions. Chitosan/TPP nanoparticles (200–400 nm with positive surface charge) have been designed for oral delivery of venlafaxine with high entrapment efficiency but a strong burst effect was observed [23]. To the best of our knowledge, the use of chitosan/TTP spray dried microcapsules to prepare controlled venlafaxine formulation has not been reported yet. In this work, chitosan/TPP microcapsules were produced by spray-drying. The effect of chitosan physico-chemical properties, TPP concentration and chitosan: TPP ratio was evaluated. The release was carried out both in simulated intestinal fluid and simulated gastric fluid.

## 2. Results and Discussion

### 2.1. Chitosan Characterization

Chitosan biological and technological properties are driven by its physico-chemical properties [10]. Therefore, one should expect different behaviors depending on the chitosan used for venlafaxine formulation. In this work, two chitosan samples with different viscosity average molecular weight (Mv) and deacetylation degree were tested as matrixes for venlafaxine formulation as seen in Table 1. Differences in molecular weight and deacetylation degree are directly related to chitosan viscosity in solution. In both samples, chitosan viscosity was appropriated for microcapsules production by spray drying.

Moreover, for pharmaceutical purposes, chitosan samples have to fulfil specific requirements related to polymer purity [24]; therefore, the ash content, which is a measurement of the inorganic material associated to the polymer, was determined. In all cases, samples with negligible or low ash content were used which indicates an appropriate polymer isolation from the crustacean shell (Table 1).

### 2.2. Microcapsules Characterization

As seen in Table 2, several formulations were developed modifying the chitosan sample, TPP concentration and chitosan-TPP ratio. These formulations were characterized in terms of practical yield (efficiency of the spray-drying process), encapsulation efficiency (EE) and surface charge (zeta potential, ζ).

At lab scale, spray-drying shows a relatively low practical yield due to the powder adhering to the cyclone walls and the small and lightest particles that are not trapped by the aspirator of the spray-dryer. Moreover, the use of aqueous solvents also accounts for lower practical yields [25]. In this work, practical yields ranging from 40 to 75% were observed. These values are in good agreement with previous lab-scale results [26]. It is worth mentioning that small volumes of the feed solution were sprayed during particle production (100 mL) which account for these low practical yields.

As seen in Table 2, the non-crosslinked formulations produced with the chitosan sample with the higher viscosity (sample CS-1) showed a higher practical yield than sample A2 produced with the lower viscosity simple (CS-2). This is in good agreement with previous results, when diltiazem hydrochloride microspheres were formulated by spray-drying [27]. In this case, the higher polymer concentration (higher solution viscosity) higher was the practical yield. On the contrary, crosslinked formulations (A3–A10) produced using chitosan sample CS1 showed a lower practical yield than those produced using sample CS2 which is an unexpected result. This may indicate that, in this system, a larger amount of lightest microcapsules that are not trapped by the aspirator of the spray-dryer were produced when using chitosan CS1 to encapsulate venlafaxine in chitosan crosslinked with TPP microspheres.

The encapsulation efficiency (EE) of the microcapsules depended to some extent on chitosan viscosity. When sample CS-1 was used, lower EE values were obtained. It has been hypothesized that an increase of chitosan viscosity hinders chitosan-TPP interactions resulting in less compact polymeric matrix which reduces the encapsulation efficiency [16]. For the same chitosan sample, the best EE was observed when 0.1% or 0.2% TPP was used. Chitosan–TPP ratio had a strong effect on EE. When the ratio was modified from 100:30 to 100:80 or 100:100 a strong reduction of the EE was observed. During microspheres production, venlafaxine is positively charged and therefore it can interact with TPP which could explain EE reduction as TPP increases in the media. Reduction of EE due to ionic interactions between TPP and the drug has been reported to occur in the preparation cross-linked chitosan microspheres for controlled release of ampicillin [28].

**Figure 1:**
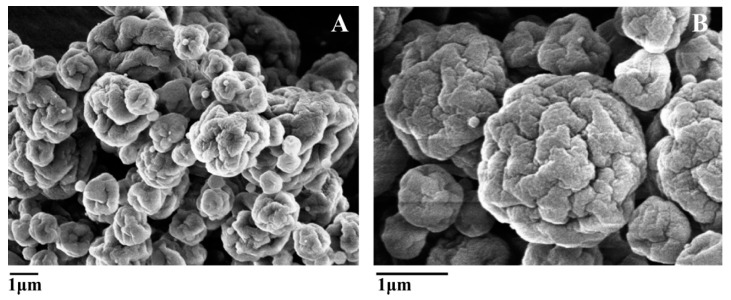
SEM of loaded microcapsules cross-linked with TPP. Sample A3 (**A**) and Sample A5 (**B**).

All microcapsules were spherical in shape with deep indentations and no venlafaxine crystals were observed on the microcapsule surface as seen in Figure 1. Particle sizes were estimated by SEM micrographs and light scattering. Microcapsules were highly polydispersed and the particle size of each formulation ranged between 3 and 10 μm with no relevant differences due to formulation composition.

**Figure 2:**
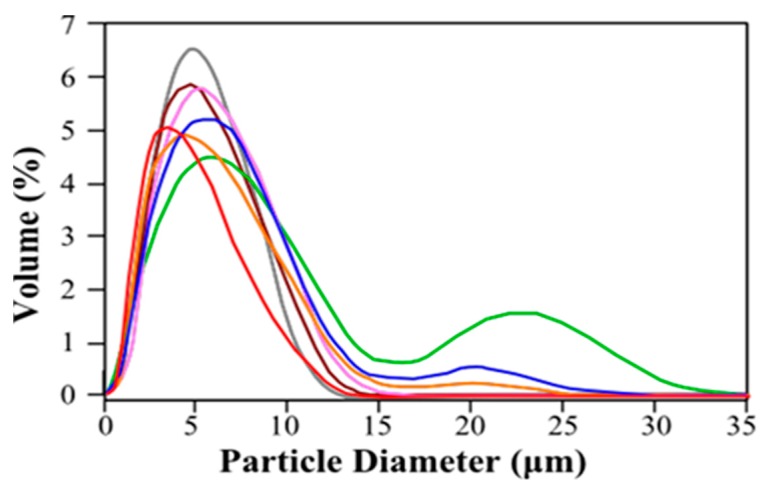
Examples of particle size measurement by light scattering. Red line; control (non-loaded microcapsules), Brown, Grey, Orange, Pink, Blue and Green lines; loaded microcapsules (A3, A6, A7, A10, A12 and A13, respectively).

The microcapsules’ size slightly increased when measured by light scattering. This can be attributed to some swelling caused by the liquid environment. Some samples exhibited bimodal distribution with a second peak centered around 20–25 μm (Figure 2). This result was not detected in SEM observations. This seems to indicate microcapsule aggregation in solution, which is to be expected from the zeta potential values of the particles (Table 2). In all cases, zeta potential values were in the range +30/−30 mV which cannot assure total repulsion of the particles. Formulations produced with a Cs-TPP ratio 100:30 showed a positive net charge that was reduced as the TPP concentration was increased. On the other hand, microcapsules produced with Cs-TPP ratios 100:100 and 100:80 were negatively charged due to the excess of negative charge provided by TPP.

Chitosan has been widely used for developing drug delivery systems because of its excellent mucoadhesive properties [20,21,22]. It has been hypothesized that this effect is due to the interaction of positively charged chitosan with negatively charged mucin from the mucus layer. Although this ionic interaction plays a fundamental role on the chitosan mucoadhesive properties, other forces such as hydrogen bonds and hydrophobic associations are also involved in this phenomenon [29]. Therefore, all formulations have potential mucoadhesive properties, with those bearing an overall positive charge being potentially more mucoadhesive.

### 2.3. Drug Physical State

**Figure 3:**
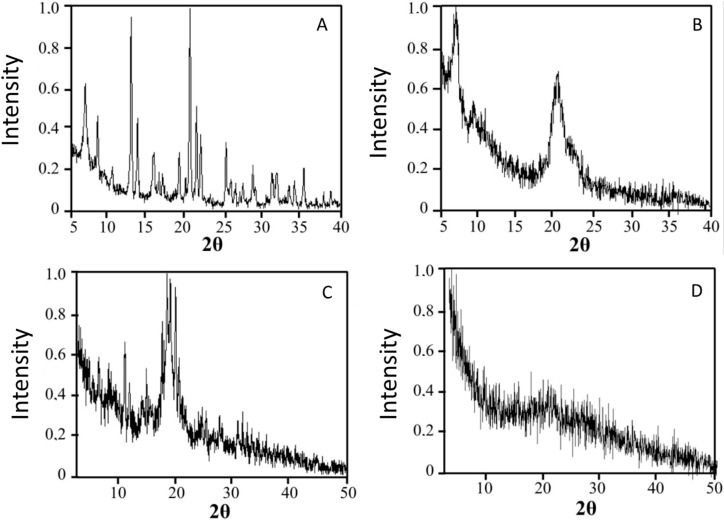
X-ray patterns of: (**A**) venlafaxine hydrochloride; (**B**) CS-1; (**C**) physical mixture of chitosan, TPP and venlafaxine hydrochloride; (**D**) chitosan-tripolyphosphate (TPP) microspheres loaded with venlafaxine hydrochloride.

Drug release kinetics from the microcapsule is affected by the physical state of the drug in the polymeric matrix; this physical state can vary from molecular dispersion (amorphous drug) to well-defined crystalline structures. XRD is a powerful tool to determine the physical state of the drug in the microcapsules. As seen in Figure 3, venlafaxine hydrochloride is highly crystalline with characteristic reflections at 6.74°, 8.38°, 12.7°, 13.14°, 16.44°, 18.98°, 20.38°, 21.22°, 21.82°, 25.1°, 28.62° and 35.14°. The presence of reflections at 8.4°, 12.8° and 16.4° and the absence of strong reflections at 10.30°, 20.30° and reflections at 15.10°, 18.30° and 22.75° pointed to the presence of venlafaxine in form II [23]. A physical mixture of venlafaxine, chitosan and TPP with the same ratios presented in the loaded microcapsules was analyzed and the more intense reflections from venlafaxine and TPP could be identified in the mixture. These reflections were completely absent when the loaded microcapsules were analyzed, indicating that venlafaxine formed a molecular dispersion in the polymer matrix. This result was in good agreement with the SEM observation where no venlafaxine crystals where seen on the microcapsules surface. The same was observed in other formulations based on chitosan where drugs were presented in molecular dispersions inside the polymer matrix [16].

### 2.4. In Vitro Drug Release in Simulated Gastric Fluid (SGF) and Simulated Intestinal Fluid (SIF)

In vitro release of the different formulations was carried out in SGF for 4 h. Although some authors have described that chitosan spray-dried microcapsules can swell and dissolve in acidic media [17], our formulations were stable for at least 8 h in the SGF media used and a controlled drug release with time was observed; even when non-cross-linked microcapsules were tested. The simulated gastric fluid used in our work was composed of HCl 0.1 M and NaCl 0.1 M as described by U.S. *Pharmacopeia* (USP)*.* The presence of NaCl in the media increases the ionic strength of the solution which may explain this difference since lower polymer swelling is expected in these conditions when compared with HCl 0.1 M solution used by other authors.

**Figure 4:**
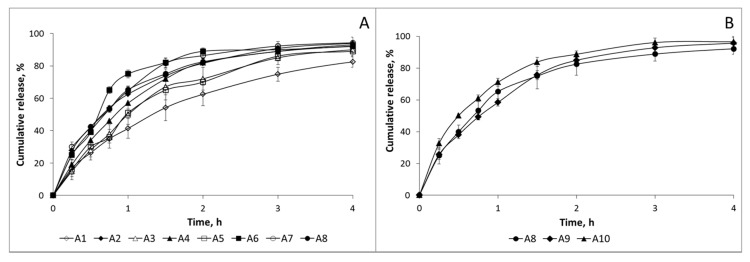
Release pattern in simulated gastric fluid (SGF) at 37 °C. (**A**) Comparison of formulations with chitosan (CS):TPP ratio 100:30; (**B**) Comparison of formulations with different CS:TPP ratio. Open symbols, chitosan CS-1; close symbols, chitosan CS-2.

As seen in Figure 4A, at low CS:TPP ratio (100:30), release was fast and more than 80% was released in the first 4 h even when high TPP concentrations were used (TPP 0.5%). A closer inspection of the release patterns showed that almost no differences were observed due to the chitosan sample used. Moreover, an increase in CS:TPP ratio did not modify the release pattern as seen in Figure 4B.

**Figure 5:**
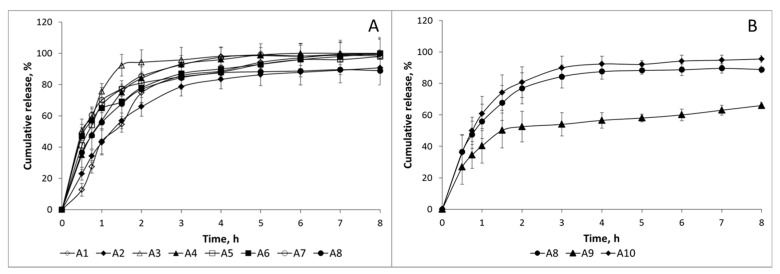
Release pattern in simulated intestinal fluid (SIF) at 37 °C. (**A**) Comparison of formulations with CS:TPP ratio 100:30; (**B**) Comparison of formulations with different CS:TPP ratios. Open symbols, chitosan CS-1; close symbols, chitosan CS-2.

In vitro release of the different formulations was also carried out in SIF for 8 h. Release patterns were also very fast, as seen in Figure 5A. In fact, release was more sustainable in SGF than in SIF in several formulations. This is in good agreement with previous results where different commercial venlafaxine formulations showed a more controlled release in acidic media (HCl 0.1 M) than in water or phosphate buffer at pH 6.8 [30]. A closer inspection of the figure reveals little effect of the chitosan sample on the release pattern observed. In contrast to the previously observed release pattern in SGF, a more controlled release was observed as the CS:TPP ratio increased (Figure 5B). In sample A10 (CS:TPP ratio 100:100), release was reduced to 60% in 8 h. In this sample, a bimodal release pattern was clearly observed; during the first 1.5 h, release was fast and after that a more sustainable release was detected. It has been reported that after oral administration of venlafaxine via oral route pharmacokinetic parameter C_max_ has been achieved in 120 min [26]. The steady state half-lives of venlafaxine and o-desmethylvenlafaxine (active metabolite of venlafaxine) are 5 and 11 h [2]; therefore, the release pattern of the formulations in which a first fast release followed by a more sustainable one is observed is favorable to keep the appropriate blood concentration of the drug in the patients. Furthermore, the presence of chitosan may enhance the permeability of the drug through the GI tract not only due to its mucoadhesive properties but also due to its ability to open the tight junctions.

The understanding of the release process is of great interest to improve drug formulations. The following phenomena are involved in venlafaxine release from chitosan based microcapsules. At the beginning of the process, steep water concentration gradients appear at the polymer/water interface causing water imbibition into the polymer matrix.

(i) Due to water imbibition, chitosan swells increasing the dimensions of the particles. This swelling is controlled by the polymer cross-linking with TPP.
(ii) Upon contact with water, venlafaxine dissolves and, due to concentration gradients, diffuses out of the microcapsules.
(iii) Depending on microcapsule composition, certain polymer dissolution in SGF is expected. Since microcapsules maintain their integrity during the release assay even in the absence of TPP this phenomenon may be negligible at least at the beginning of release.

Taking into account these phenomena, the venlafaxine release from the chitosan based microspheres must be governed by several processes. In other to get insight in the release mechanism data were fitted to the Peppas-Korsmeyer equation (Equation (1)) [31].

(1) $$\frac{Mt}{M\infty }=K\times t^{n}$$

where *K* is the kinetic constant and *n* is an exponent characterizing the diffusional mechanism. The equation becomes physically realistic in two cases. When *n* = 0.43 (pure diffusion controlled drug release) and *n* = 0.85 (swelling-controlled drug release or Case II transport) for spherical geometries. Other values of *n* between 0.43 and 0.85 indicate anomalous transport. This semi-empirical equation is usually used for the analysis of the first 60% of the release curves. However, it has been proposed that this equation can be used to fit the ‘entire’ drug release curve, at least in drug delivery systems based on hydroxypropyl methylcellulose [32]. With this completed fit, a release mechanism based on the non-classical diffusion of the solute within the polymeric system has been proposed.

The values of n after fitting the data to the Peppas-Korsmeyer equation both in SGF and SIF using the first 60% of the release curve are shown in Table 3. Attempts to fit the entire drug release curve to the Peppas-Korsmeyer equation were unsuccessful.

**Figure 6:**
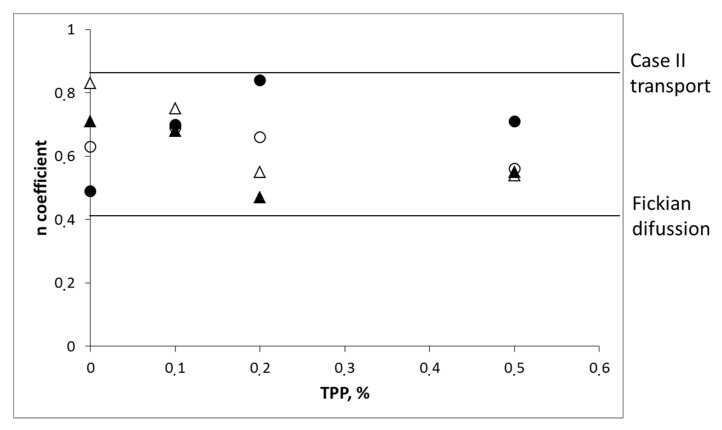
*n* values of the Korsmeyer–Peppas equation vs. TPP concentration. Formulations based on CS-1 (open symbols). Formulations based on CS-2 (close symbols). Simulated gastric fluid (circles) and simulated intestinal fluid (triangles).

In simulated gastric fluid, values of n between 0.49 and 0.84 were observed which indicated a combined mechanism of pure diffusion and Case II transport (Figure 6). In simulated intestinal fluid, all samples except sample A10 showed values of n between 0.47 and 0.75, again indicating a combination of processes. In sample A10, a value of n lower than 0.45 was observed. Deviations from 0.45 values have been previously reported by other authors and interpreted in terms of polydispersion [33] and heterogeneity of the polymer layer [34].

## 3. Materials and Methods

### 3.1. Materials

Chitosan sample labelled as CS-1 was purchased from Primex (Siglufjordur, Iceland) and chitosan sample CS-2 was kindly donated by Idebio S.L. (Salamanca, Spain). Venlafaxine hydrochloride (Uquifa México, S.A., Morelos, Mexico, batch number: 1040030001) was kindly donated by Vegal S.L. (Spain). Acetic acid and pentasodium tripolyposphate (analytical grade) were purchased from Sigma-Aldrich (St. Louis, MO, USA). Ultrapure Milli-Q water was used throughout. Other reagents were of analytical grade.

### 3.2. Methods

#### 3.2.1. Chitosan Deacetylation Degree Determination

Chitosan deacetylation degree (DD) was determined by UV-spectroscopy [35]. The zero crossing point (ZCP) was determined by superimposing the first derivative spectra of 0.01, 0.02 and 0.03 M of acetic acid solutions at 203 nm. Solutions of 0.005–0.05 mg of GlcNAc per mL of 0.01 M acetic acid solution were prepared and their first derivative spectra obtained (from 190 to 240 nm). The vertical distance from ZCP to each GlcNAc solution spectrum, H, was measured (mm). A linear calibration curve was obtained by plotting the H values against the corresponding GlcNAc concentration. Chitosan samples were dissolved at 0.1 mg/mL in acetic acid 0.1 M and the UV-spectra were recorded between 190 and 240 nm. The H values of the chitosan samples were measured and the contribution due to GlcNAc was obtained from the calibration curve. The DD of the samples were determined by the formula (Equation (2)):

(2) $$DD\;\left(\%\right)=\left(100-\frac{\left[NAG\right]}{\left[Cs\right]}\right)\times 100$$

#### 3.2.2. Determination of Intrinsic Viscosity ([η]) and Molar Mass Estimation of Chitosans

Chitosan intrinsic viscosity was determined in 0.3 M AcOH/0.2 M AcONa at 25 °C [36]. Samples with a chitosan concentration ranged from 0.1 and 0.07 mg/mL were used for the determinations. The relative viscosity was measured using an Ubbelohde Capillary Viscometer type 525/13. Each determination was carried out in triplicate. The Mark-Houwink-Sakurada relationship was used to determine the viscosimetric mean average molar mass (Equation (3)):

(3) $$\left[\eta \right]=K\times M^{\alpha }$$

where *K* was estimated to be 7.4 × 10^−2^ and 7.6 × 10^−2^ (mL/g) for CS-1 and CS-2, respectively, taking into account their acetylation degree values and α was 0.76 in both cases [35].

#### 3.2.3. Microcapsules Production

Microcapsules were produced by spray drying using a Büchi Mini Spray Dryer B-290 (Flawil, Switzerland) with a standard 0.5 mm nozzle. Chitosan (0.5% *w*/*v*) was dissolved in acetic acid 1% (*v*/*v*) and venlafaxine was added to this solution at 30% (*w*/*w*) with respect to chitosan at constant stirring until homogenization. TPP (0.1% to 0.5% in water) was then added under gentle stirring during 30 min to the venlafaxine–chitosan solution in variable ratio (100:30; 100:80 or 100:100). Opalescence appeared during TPP addition but no aggregates were observed. The chitosan–TPP–drug suspension was then spray dried to obtain the TPP cross-linked chitosan microspheres loaded with venlafaxine. Spray dried conditions were set as follows: flow rate 32 m^3^/h, compressed air flow rate 473 NL/h and inlet temperature 160 °C. Different formulations were produced by modifying the type of chitosan sample, TPP concentration and chitosan/TPP ratio.

#### 3.2.4. Spray Drying Production Yield

The efficiency of the spray drying process was gravimetrically determined using Equation (4)

(4) $$Practical\;yield=\frac{total\;recovey\;weight\;after\;spray-drying,\;g}{Theorical\;weight,\;g}\times 100$$

where theoretical weight is the sum of the chitosan, TPP and venlafaxine weights in the feed and total recovery is the powder weight after the spray-drying process

#### 3.2.5. Encapsulation Efficiency

To determine the encapsulation efficiency (EE) a known amount of microcapsules were crushed in a glass mortar and digested in HCL 0.1 N at 37 °C under strong mechanical stirring (1600 rpm) during 24 h. The samples were centrifuged at 20,000 rpm (Hettich Centrifugen micro, Herrenberg, Germany) for 3 min and the absorbance was determined at 225 nm. Preliminary studies showed that no polymer interference occurs at this wavelength. A calibration curve was prepared by dissolving venlafaxine in HCL 0.1 N in appropriate concentrations (r = 0.995).

EE was determined using the Equation (5).

(5) $$EE=\frac{VEN\;detected\;after\;microcapsules\;disruption,\;mg}{VEN\;added\;to\;microcapsules,\;mg}\times 100$$

#### 3.2.6. Shape and Morphology Determination

The shape and surface of the microcapsules were observed under scanning electron microscopy (SEM) with a JEOL JSM-6400 microscope (Jeol, Tokyo, Japan) at 10 kV accelerating voltage. The samples were previously sputter coated with Au/Pd using a vacuum evaporator (Balzers SDC 004 Sputter coater, Oerlikon Corporate Pfaffikon, Altendorf, Switzerland).

#### 3.2.7. Size Distribution

The size distribution of the microcapsules was determined by light scattering (Coulter^®^ LS 230, small volume module, Beckman Coulter Inc., Indianapolis, IN, USA). Samples were suspended in ethanol (Refractive index 1.359) to minimize their swelling.

#### 3.2.8. Zeta Potential

Surface charge of the microcapsules (zeta potential) was estimated by microelectrophoresis using a Malvern Zetasizer Nano ZS (Malvern Instruments, Herrenberg, Germany). Measurements were carried out at an effective voltage of 150 V and 25 °C. Electrophoretic mobility data were automatically converted using the Henry equation and the Helmholzt-Smoluchowski approximation [37]. All measurements were recorded in triplicate.

#### 3.2.9. X-ray Diffraction (XRD) Studies

The physical state of venlafaxine in the microcapsules was assessed by XRD. X-ray powder diffraction spectra of pure venlafaxine, pure TPP, chitosan (CS-1), loaded microcapsules and a physical mixture of chitosan, TPP, and venlafaxine hydrochloride in the same weight ratio as loaded microcapsules were recorded using a PHILIPS X’PERT MPD diffractometer (Philips, Amsterdam, The Netherlands), using Cu Kα, radiation operated at a voltage of 40 kV. The samples were analysed in 2-teta angle range 5–50. The process parameters were set at as scan step size 0.040°, scan step time of 1 s, room temperature.

#### 3.2.10. In Vitro Release of Venlafaxine

In vitro release test were carried out in simulated gastric fluid (SGF; pH 1.2 HCl 0.1 M/NaCl 0.1 M) and simulated intestinal fluid (SIF; pH 7.4 KH_2_PO_4_ 0.05 M/NaOH 0.04 M) without enzyme as described by USP 23 [37]. 25 mg of microcapsules were suspended in the release medium inside a cellulose dialysis bag (dialysis tubing MW cut-off 12 kDa, Sigma-Aldrich, St. Louis, MO, USA) to avoid the loss of microcapsules during the test. Then the dialysis bag was placed in 250 mL of the release medium at 37 °C with rotational stirring (100 rpm). At pre-determined time intervals, samples were withdrawn (5 mL) and the same volume of tempered fresh media was added. Previous studies indicated the dialysis membrane has negligible effect in the release kinetics and all the release is carried out in sink conditions. The amount of released venlafaxine was determined spectrophotometrically at 225 nm. Calibration curves were prepared by dissolving venlafaxine in SGF or SIF, respectively, in appropriate concentrations (r = 0.995). In vitro release studies were performed in triplicate.

The in vitro release profiles were fitted to Peppas-Korsmeyer model (Section 2.4) using solver tool from Microsoft Excel (Redmond, WA) and the release exponent was determined. The goodness of the fits was evaluated with the sum of squared deviations (SSD). The sum of squared deviations was calculated with the following equation:

SSD = ∑ (Y_experimental_ − Y_model_)^2^.

#### 3.2.11. Statistical Analysis

Values are expressed as mean ± standard deviation. Mean and standard deviation of the results from at least three independent experiments were calculated using Microsoft Excel (Redmond, WA, USA) software.

## 4. Conclusions

In this paper, a highly soluble drug (venlafaxine) was formulated, for the first time, in chitosan-based microspheres to produce controlled release formulations. The effect of chitosan physico-chemical properties, TPP concentration and chitosan-TPP ratio on drug release was evaluated. Chitosan physico-chemical properties have some effect on the practical yield and encapsulation efficiency but little effect on drug release pattern. The parameter with the main effect on venlafaxine release was chitosan-TPP ratio.

A formulation based on chitosan with low viscosity and a chitosan:TPP ratio of 1:1 showed the most moderate controlled release, with a maximum release at 6 h in SIF of 60%.

## Figures and Tables

**Table 1:** Chitosan characterization.

Sample Name	Source	Ash Content, %	(η), dL/g	Mv, kDa	DD, %
CS-1	Blue Crab (*Callinectes* sp.)	0.01	1.975	644	90.5
CS-2	King Crab (*Paralomis granulosa*)	<0.01	1.171	324	83.8

Mv: Viscosity Average Molecular Weight; DD: Deacetylation degree.

**Table 2:** Microcapsules formulation and main characteristics.

Formulation Code	CS Sample	[TPP], %	CS:TPP	Practical Yield, %	EE, %	ζ, mV
A1	CS-1	0	-	63	56 ± 3	+26.6
A2	CS-2	0	-	53	88 ± 3	+19.1
A3	CS-1	0.1	100:30	44	81 ± 2	+21.5
A4	CS-2	0.1	100:30	65	94 ± 1	+16.6
A5	CS-1	0.2	100:30	45	76 ± 3	+13.4
A6	CS-2	0.2	100:30	62	90 ± 3	+16.6
A7	CS-1	0.5	100:30	55	51 ± 5	+1.3
A8	CS-2	0.5	100:30	62	66 ± 3	−0.8
A9	CS-2	0.5	100:80	64	37 ± 3	−5.7
A10	CS-2	0.5	100:100	74	38 ± 4	−9.4

EE: Encapsulation efficiency; ζ: zeta potential. Cs concentration 0.5% *w*/*v*.

**Table 3:** Parameter estimation derived from the fitting of experimental data to Peppas-Korsmeyer equation using solver tool from Excel.

Formulation Code	*n* SGF	SSD	*n* SIF	SSD
A1	0.63	0.00027	0.83	0.007998
A2	0.49	0.00246	0.71	0.001473
A3	0.69	0.00576	0.75	0.000464
A4	0.70	0.00093	0.68	0.000154
A5	0.66	0.00629	0.55	0.002309
A6	0.84	0.00415	0.47	0.000002
A7	0.56	0.00028	0.54	0.000001
A8	0.71	0.00001	0.55	0.000292
A9	0.60	0.00007	0.62	0.000857
A10	0.55	0.00328	0.32	0.003435

SSD: Sum of squared deviations (SSD = ∑ (Y_experimental_ − Y_model_)^2^.
